# Pain Complaints in Patients Undergoing Orthognathic Surgery

**DOI:** 10.1155/2018/4235025

**Published:** 2018-07-15

**Authors:** Jimoh Agbaje, Jonathan Luyten, Constantinus Politis

**Affiliations:** ^1^OMFS-IMPATH Research Group, Department of Imaging and Pathology, KU Leuven, Leuven, Belgium; ^2^Department of Oral Health Sciences, KU Leuven, Leuven, Belgium; ^3^Oral and Maxillofacial Surgery, Leuven University Hospitals, Leuven, Belgium

## Abstract

The aim of this retrospective study was to assess the frequency of orofacial (nonodontogenic, neuropathic, or atypical) and temporomandibular joint (TMJ) and/or masticatory muscle pain in orthognathic patients in a tertiary institution. A total of 286 consecutive patients undergoing sagittal split osteotomy from 2014 to 2016 were included. Thirty-nine (13.6%) patients presented with TMJ pain and 10 (3.5%) with orofacial nonodontogenic pain before orthognathic surgery; 79.6% (39/49) of these patients had no pain 1 year after surgery. Twenty-nine patients (12.2%) with no preoperative orofacial pain and 22 (9.3%) without preoperative TMJ pain presented with pain 1 year after surgery. Fifty-one (17.8%) of the 286 patients treated for orthognathic cases at our center over the 3-year period presented with pain 1 year after surgery. Most patients were managed conservatively with nonsurgical therapeutic modalities including counseling, physical therapy, warmth application, and bilateral chewing and splint therapy. In patients with TMJ pain refractory to conservative treatment, arthroscopic surgery was advised and successful in all patients for both pain reduction and improvement of the maximal interincisal opening. TMJ symptoms do develop after orthognathic surgery in patients with and without a previous history of TMJ complaints. Most patients can be managed with nonsurgical therapeutic modalities.

## 1. Introduction

Orthognathic surgery corrects dentofacial deformity and a wide range of minor and major skeletal and dental irregularities. Severe malocclusions are associated with aesthetic impairment, functional problems, and temporomandibular joint (TMJ) and orofacial pain [1, 2]. Malocclusion puts excessive strain on the TMJ and increases the risk of developing TMJ disorder, jaw stiffness, chronic headaches, and impaired oral function [1, 2].

TMJ and muscle disorders (TMDs) are a group of conditions that cause pain and dysfunction in the TMJ and the muscles that control jaw movement [3]. The most common symptom is pain, particularly in the masticatory muscles and/or TMJ. Other likely symptoms include radiating pain in the face, jaw, or neck; jaw muscle stiffness; limited movement or locking of the jaw; painful clicking, popping, or grating in the TMJ when opening or closing the mouth; and a change in the way the upper and lower teeth occlude [3]. In some cases with major skeletal jaw discrepancies, orthodontic treatment is indicated in conjunction with orthognathic surgery to correct the occlusal relationship.

Orthognathic surgery may have a beneficial effect on TMJ disorders and orofacial pain in subjects with preexisting signs and symptoms [4]. However, the extensive bone and muscle manipulation and various movements during surgery may result in postoperative pain, especially in the TMJ. Positional changes of the mandible, maxilla, or both jaws during orthognathic surgery can affect the TMJ, masticatory musculature, and surrounding soft tissue [5, 6]. The position of the condyles in relation to the temporal bone can also be altered during surgery. Furthermore, trauma (major or due to dental procedures) can result in neuropathic pain [7]. Inferior alveolar nerve injury may occur during surgery and give rise to unpleasant sensations (e.g., allodynia, dysesthesia, and paresthesia) and, in some patients, continuous aching in the lower face (i.e., hyperalgesia and neuralgia) [8]. Known orofacial pains include maxillary sinusitis after Le Fort I osteotomies and infections or sensitivity due to the osteosynthesis material (plates and screws).

The influence of orthognathic surgery on the symptoms of TMD has been widely debated in the literature and among oral and maxillofacial surgeons, not only because of the possibility of improvement through the correction of deformities but also because of the possibility of symptom development. The aim of this study was to assess the frequency of orofacial (nonodontogenic, neuropathic, or atypical), myalgia, and TMJ pain in orthognathic patients at a tertiary institution. Our hypothesis was as follows: orthognathic surgery may induce TMJ pain and myalgia in patients without pain before treatment as well as orofacial pains.

## 2. Materials and Methods

### 2.1. Patients

Preoperative and follow-up data were collected from the records of consecutive orthognathic surgical patients between January 2014 and December 2016 with a minimum 1 year of follow-up after surgery. The primary outcome measure was the presence of self-reported nonodontogenic, neuropathic, or atypical orofacial pain and or pain in the TMJ and/or masticatory muscles (myalgia) before the surgery and during follow-up. These self-reports were categorized by the assessor as an absence of pain, the presence of orofacial nonodontogenic pain, the presence of neuropathic or atypical orofacial pain, the presence of masticatory muscle and/or TMJ pain, normal sensation, and altered sensation (hypoesthesia, anesthesia, or dysesthesia). The diagnosis of pain and altered sensation was based primarily on a questionnaire, visual analog scale (VAS) pain score, history, and findings on physical examination [9].

This retrospective study was approved by the Medical Ethical Committee of Leuven University Hospitals under number S57587.

### 2.2. Surgery

The bilateral sagittal split osteotomy (BSSO) surgical technique was described in detail elsewhere [10, 11]. All surgical procedures were done by the same surgical team. The main surgeon's experience included more than 4000 orthognathic procedures spanning a period of over 25 years. Only those orthognathic procedures were included in which also a sagittal split osteotomy of the lower jaw was performed. Orthognatic procedures in syndromic patients were excluded.

### 2.3. Pain Variables

Pain was assessed the same way in all patients. General information about the pain complaint was obtained using a pain questionnaire and complimentary interview. The questionnaire was designed to obtain as much basic information about the pain as possible within the limits of brevity, clarity, and self-administration. This information was completed during the interview. All patients were interviewed before orthognathic surgery and during the follow-up period by trained surgeons using a standardised protocol. The clinical pain assessment evaluated the presence or absence of pain. The subjective pain intensity present with the jaw at rest was assessed using a VAS. Subsequently, an increased pain response to voluntary opening and closing, as well as to moving the jaw anteriorly and laterally, was recorded. During subsequent assessments, joint tenderness was scored as either absent or present. The presence of muscle tenderness was assessed during mouth opening and closing and by digital palpation. Muscle tenderness was scored as being either present or absent. The presence or absence of pain and altered sensation was also noted. All patients with pain were managed postoperatively. Acute pain was managed with analgesia, such as nonsteroidal anti-inflammatory drugs (NSAIDs). Benzodiazepines were also used to manage acute TMJ pain accompanied by limited opening. Conservative modalities such as counseling, physical therapy, warmth application, and bilateral chewing and splint therapy were also applied. Osteosynthesis plate removal was done in patients with pain secondary to osteosynthesis plate. In patients with TMJ pain refractory to conservative treatment, arthroscopic surgery was advised.

### 2.4. Data Collection

Information on patient age, gender, presence or absence of pain, presence of altered sensation, and type of surgery were recorded.

## 3. Results and Discussion

### 3.1. Results

The study included a total of 286 patients (102 males, 184 females) aged 16–66 years (mean age 26.7 ± 12.3 years). During the 3-year study period, all patients who presented at the maxillofacial unit of UZ Leuven Hospital for orthognathic surgery were examined and followed up for a minimum of 1 year after surgery. One hundred twenty-two (42.66%) interventions required bimaxillary osteotomies, and 164 (57.34%) required bilateral sagittal split osteotomies (BSSOs). There was no report of altered sensation and muscle tenderness in any of the patients preoperatively, but 36 patients presented with pain associated with TMJ (most patients in this group complain of discomfort and sometimes pain when chewing or opening the mouth, while some present with nonpainful sound when opening the mouth), 7 patients had orofacial nonodontogenic pain, and 3 patients complained of both TMJ and orofacial nonodontogenic pain. Preoperatively in most of the patients, the pain complaint apart from mild discomfort has little effect on their daily activity. The pain was reported to be intermittent in some patients, whereas others reported pain from time to time; the recorded VAS score ranged from 1 to 3 in patients with pain. The number of patients complaining of orofacial pain and TMJ pain decreased during follow-up (Table 1). Of the 13.6% of patients presenting with TMJ pain and 3.5% presenting with orofacial nonodontogenic pain before orthognathic surgery, 79.6% (39/49) had no pain 1 year after surgery. Approximately 80% (8/10) of the patients with orofacial pain and 79.5% (31/39) of the patients with TMJ pain before surgery were pain-free 1 year after surgery.

**Figure 1:**
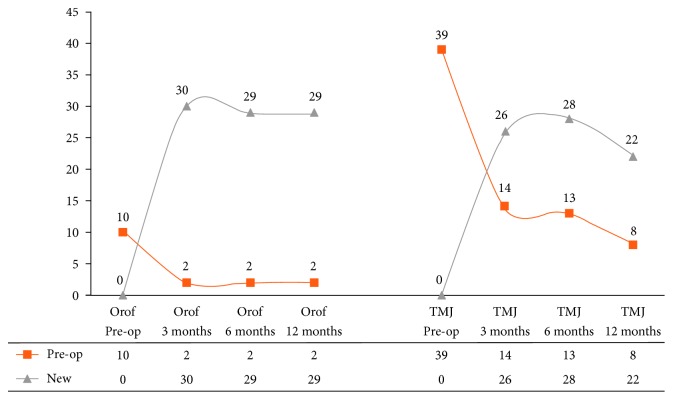
Overview of patients with pain before and during the follow-up period. Orof, orofacial; Pre-op, preoperative.

An overview of patients with pain (orofacial and TMJ) before and during the follow-up period is presented in Figure 1. Twenty-nine (12.2%) patients with no preoperative orofacial pain and 22 (9.3%) without preoperative TMJ pain presented with pain 1 year after surgery. Five of these patients (2.1%) complained of both orofacial and TMJ pain (Table 1).

**Figure 2:**
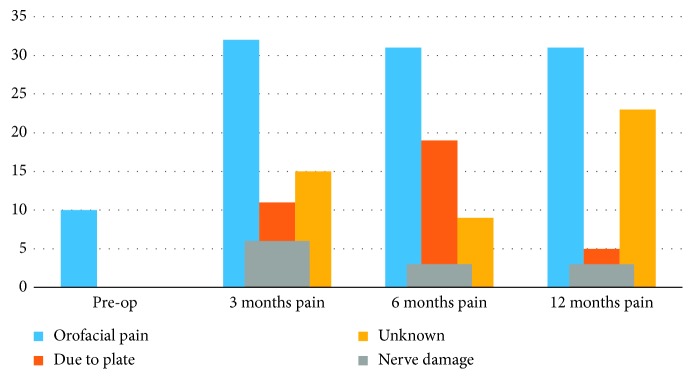
Common causes of nonodontogenic orofacial pain (TMJ pain not included).

Fifty-one (17.8%) of the 286 orthognathic cases treated at our center over the 3-year period presented with pain 1 year after surgery; 3 (1.04%) were nerve related, 5 (1.75%) were due to osteosynthesis plates, 23 (8.04%) atypical odontalgia, and 30 (10.50%) were TMJ related. There are 28 patients (20 female and 8 male) with new TMJ pain with an average age of 30 years. The common causes of nonodontogenic orofacial pain in the patients are shown in Figure 2.

Pain was managed with medication such as nonsteroidal anti-inflammatory drugs, conservative modalities such as physical therapy, osteosynthesis plate removal, and arthroscopic surgery in TMJ pain refractory to conservative treatment.

### 3.2. Discussion

Our findings are in agreement with those reported by Dujoncquoy et al. [12] and Westermark et al. [13], who showed that patients with preexisting TMJ dysfunction undergoing orthognathic surgery are likely to have significantly improved signs and symptoms of TMJ dysfunction after surgery. Borstlap et al. also reported the disappearance of signs and symptoms of TMJ dysfunction in 56% of the 87 patients who had preexisting signs and symptoms before surgery, whereas 22% of the 135 patients without preexisting symptoms of TMJ dysfunction developed TMJ dysfunction [14, 15]. The 80% improvement in TMJ symptoms observed in this study is close to the 89.1% improvement observed by White and Dolwick [16], but less than the improvement reported by other authors (40–66%) [17–19]. The observed differences in the percentage of patients who improved after surgery can be due to the type of patient population involved and the number of cases included in the study, as a patient's skeletal deformity could have a direct impact on TMJ symptoms after surgery [20, 21].

Orthognathic surgery in the lower jaw strains the condylar area during surgical maneuvers in the ascending ramus. After orthognathic surgery, most patients heal without symptoms or signs at the TMJ, with a functional occlusion. However, reproducing the original condylar position is difficult, and too much pressure can be placed against the articular disc or an unfavorable condylar position can be created during osteotomy, which can worsen any preexisting TMJ symptoms or result in new ones [12]. Hu et al. [18] and White and Dolwick [16] previously reported a new TMJ symptom rate of 8% and 8.1%, respectively, after orthognathic surgery, compared to the 8.9% of patients with new TMJ pain and 11.7% with orofacial pain in the present study. Though the causes of new TMJ symptoms in patients without preoperative symptoms are not known, one of the hypotheses proffered is the mechanical overload exerted on both the condyle and disc during manual repositioning of the ascending ramus in centric relation and during osteosynthesis, which results in excessive compression of the disc-condyle complex towards the posterior slope of the fossa, or from excessive rotation or tilting of the condyle due to bony interference between the proximal and distal bone fragments in sagittal split osteotomies, and/or from compression force elicited from osteosynthesis plates or screws. These situations can potentially result in joint noise or pain and can worsen any preexisting TMJ symptoms [12, 22–24].

TMJ pain can also be attributed to dysfunctional TMJ remodeling, which can be induced by systemic and local arthritis or trauma [20, 21] The average age of patients with TMJ pain in this study was approximately 30 years, which is consistent with the tendency of TMJ pain to be age-related, as it is commonly found in young and middle-aged adults [25–27].

We observed that the majority of patients with the new TMJ pain are women. Of the 28 new TMJ cases, 20 (71.4%) were female, which could be related to the previous findings by Aghabeigi et al. that women with an atypical psychological profile seem to be at increased risk of persistent postoperative TMJ pain after orthognathic treatment and open bite deformity [28].

Osteotomy has been known to induce changes in the maxillary and mandibular teeth, buccal mucosa, palatal mucosa, and facial skin sensation. Though skin sensation tends to recover over time, even after direct damage to the sensory nerves, it may not completely recover to the presurgical condition. Postoperative orofacial pain includes a number of clinical problems involving the masticatory muscles or TMJ, including muscle spasms in the head, neck, and jaw and pain in the teeth, face, or jaw due to the extensive bone and muscle manipulation during orthognathic surgery [29].

Neurological injuries during orthognathic surgery due to an exposed nerve together with disruption of the bony environment of the nerve may result in orofacial and/or musculoskeletal pain [6, 30, 31].

Early diagnosis in conjunction with patient education is central to the management of orofacial and TMJ pain. The first line management of TMJ pain consists of conservative measures. Medications may be considered to manage the symptoms associated with pain in conjunction with patient education [6]. Medical management following the NICE guidelines for neuropathic pain may also be implemented [32]. All patients in the present study were managed conservatively with nonsurgical therapeutic modalities focusing on patient education to encourage the development self-management strategies [33]. Nonsurgical therapeutic modalities including counseling, physical therapy, warmth application, and bilateral chewing were encouraged. Occlusal splints were also used to alleviate TMJ symptoms [34].

Acute TMJ pain is managed effectively with simple analgesia, such as nonsteroidal anti-inflammatory drugs (NSAIDs) [35]. Benzodiazepines are also used to manage acute TMJ pain accompanied by limited opening [36]. TCAs, such as amitriptyline or nortriptyline, may be effective in managing chronic TMJ pain refractory to conservative measures [36].

In patients without preexisting TMJ complaints in whom orthognathic surgery resulted in TMJ complaints refractory to conservative treatment, arthroscopic surgery is advised, as it seems to be successful in all patients for both pain reduction and improvement of the maximal interincisal opening [37]. However, surgical interventions involving the TMJ should be avoided in patients with chronic TMJ complaints without significant functional impairment, as such interventions are unlikely to yield benefits and may exacerbate their symptoms [34].

## 4. Conclusions

Approximately 80% of the patients with orofacial pain and TMJ pain before orthognathic surgery were pain-free 1 year after orthognathic surgery. While 12.2% of patients with no preoperative orofacial pain and 9.3% without preoperative TMJ pain presented with pain 1 year after orthognathic surgery.

## Figures and Tables

**Table 1:** Number of patients with orofacial and TMJ pain before surgery and during the follow-up period (*N* = 286).

Type of pain	Cases	Period (months)			
		0	3	6	12
Orofacial pain	Existing	10	2	2	2
	New	0	30	29	29
	Total	10	32	31	31

TMJ pain	Existing	39	14	13	8
	New	0	26	28	22
	Total	39	40	41	30

Patients without orofacial and TMJ pain		237	214	214	225

## Data Availability

The patient data used to support the findings of this study are restricted by the Medical Ethical Committee of Leuven University Hospitals in order to protect patient privacy. Data are available from the corresponding author for researchers who meet the criteria for access to confidential data.
